# Whole genome sequence of *Fusarium graminearum* strain mFG23 isolated from maize ear rot

**DOI:** 10.1128/mra.00865-25

**Published:** 2025-11-10

**Authors:** Zhenyu Zhang, Luyao Duan, Wenyi Li, Haiyan Zhang, Chengjia Zou, Yan Wen, Lin Li, Li Xie, Xiao Li, Lina Cui, Weili Cai

**Affiliations:** 1 Institute of Plant Protection, Sichuan Academy of Agricultural Sciences, Key Laboratory of Integrated Crop Pest Management in Southwest China, Ministry of Agriculture, Chengdu, China; 2 Seed Station of Sichuan Province, Chengdu, China; University of California Riverside, Riverside, California, USA

**Keywords:** *Fusarium graminearum*

## Abstract

*Fusarium graminearum* causes significant disease in crops. We report the whole-genome sequence of strain mFG23, obtained from maize kernels with ear rot in Yunnan Province, China. This genome is a valuable resource for studying the genetic basis of *F. graminearum* pathogenicity and host specificity in maize.

## ANNOUNCEMENT

*Fusarium graminearum* causes Gibberella ear rot and stem rot in maize (1). The pathogen produces harmful mycotoxins that contaminate food and feed, posing serious health risks (2). In this study, we present the whole-genome sequence of *F. graminearum* strain mFG23, which was obtained from maize kernels exhibiting ear rot symptoms in Yunnan Province, China. Single-spored isolates of *F. graminearum* were obtained from surface-sterilized, infected maize kernels on potato dextrose agar (PDA) and confirmed by internal transcribed spacer (ITS) sequencing. To elucidate the genetic basis of *F. graminearum* aggressiveness in maize ear rot, we employed high-throughput sequencing using the MGI-T7 platform in combination with MGI Cyclone Nanopore long-read technology. This integrated approach enabled the generation of a high-quality scaffold-level genome assembly of strain mFG23. The newly assembled genome provides a valuable resource for comparative genomics and functional studies of *F. graminearum* in maize.

Genomic DNA was extracted from mycelial biomass that was cultured on PD media with E.Z.N.A. Plant & Fungal DNA Kit (Omega Biotek, Omega, GA). Genomic DNA was quantified by TBS-380 fluorometer (Turner BioSystems Inc., Sunnyvale, CA). A total of 2 µg of the high-quality DNA (OD260/280 = 1.8 ~ 2.0) was used to construct a paired-end sequencing library with TruSeq DNA nano library prep kit (Illumina, CA). The library was sequenced using the MGI-T7 platform (Shanghai BIOZERON Biotech. Co., Ltd) with PE150 mode. For MGI Cyclone Nanopore sequencing, 20 k insert whole genome shotgun libraries were generated with CycloneSEQ 24 Barcode Library Prep Set (MGI Tech., Shenzhen) and sequenced on G400-ER MGI Cyclone Nanopore instrument for 96 h (Flowcell version: WuTong_Sensor_beta V2.30, BGI Copyrights@2024 Jan).

A total of 8118.1 Mb raw reads with 450 bp insert size were generated from MGI-T7 sequencing. A total of 8051.4 MB clean paired-end reads were obtained by trimming and quality control with Trimmomatic (3). A total of 4.12M reads with 2,549 bp average length were generated from MGI Cyclone Nanopore sequencing. Bases were directly called by the G400-ER MGI Cyclone instrument’s software CycloneMaster. Before assembly, GenomeScope 2.0 was applied to get an estimate genome size of 37.1 Mb using 21-mers (4). Canu v2.2 was utilized for the pure long reads assembly with default parameter: correctedErrorRate = 0.144 (5). GapCloser v1.12 software was subsequently applied to fill up the remaining local inner gaps and correct the single base polymorphism (6). The software MaSuRCA v4.0.8 was used to perform hybrid assembly as follows: short reads were first assembled into super-reads, which were then aligned to extend long reads into mega-reads; Celera Assembler was used to assemble the mega-reads, followed by redundancy removal to produce the final assembly (7). The software MUMmer v4.0 was used to integrate the assembly results from MaSuRCA and Canu (8). The integrated genome sequence underwent three rounds of polishing using Racon v1.4.13, followed by at least three rounds of base correction using Pilon v1.24, ensuring a final assembly accuracy of approximately 99.9% (9, 10). The final assembly achieved a mean coverage of 200× and consists of five scaffolds, four of which are chromosomal and one mitochondrial (N50 length 9,114,578 bp). BUSCO 2.0 analysis was performed using the fungi_odb10 database to assess the genome completeness and assembly quality (11) (Table 1).

**TABLE 1:** Metrics of the *Fusarium graminearum* isolate mFG23 genome assembly

Description	Value
Assembled genome size (bp)	37,049,565
Number of scaffolds	5
Scaffold N50 (bp)	9,114,578
Scaffold 1 size	11,884,906
Scaffold 2 size	9,114,578
Scaffold 3 size	8,052,711
Scaffold 4 size	7,899,375
Scaffold 5 size (Mito）	97,995
Genome GC content (%)	47.92
BUSCO completeness (%)	99.20%
Complete and single-copy BUSCOs (S)	752 (99.20%）
Complete and duplicated BUSCOs (D)	0
Fragmented BUSCOs (F)	2 (0.30%）
Missing BUSCOs (M)	4 (0.50%）
Total BUSCO groups searched (n)	758 (100%）

## Data Availability

The raw sequencing data have been deposited in the Sequence Read Archive (SRA) database under BioSample accession number SAMN49376108 and BioProject accession number PRJNA1279076. This whole-genome shotgun project has been deposited at GenBank under accession number PRJNA1279076JBPWNR000000000. The version described in this paper is the first version, JBPWNR000000000.
